# A strategy design based on antibiotic‑resistance and plasmid replicons genes of clinical Escherichia coli strains

**DOI:** 10.1080/21655979.2022.2047543

**Published:** 2022-03-08

**Authors:** Junyan Liu, Xin Lin, Thanapop Soteyome, Yanrui Ye, Dingqiang Chen, Ling Yang, Zhenbo Xu

**Affiliations:** a College of Light Industry and Food Science, Guangdong Provincial Key Laboratoryof Lingnan Specialty Food Science and Technology, Innovation Research Institute of Modern Agricultural Engineering, Zhongkai University of Agriculture and Engineering, Guangzhou, 510225, China; b Key Laboratory of Green Processing and Intelligent Manufacturing of Lingnan Specialty Food, Zhongkai University of Agriculture and Engineering, Guangzhou, 510225, China; c School of Food Science and Engineering, Guangdong Province Key Laboratory for Green Processing of Natural Products and Product Safety, Engineering Research Center of Starch and Vegetable Protein Processing Ministry of Education, South China University of Technology, Guangzhou, China; d Home Economics Technology, Rajamangala University of Technology Phra Nakhon, Bangkok, Thailand; e School of Biology and Biological Engineering, South China University of Technology, Guangzhou, Guangdong, China; f Department of Laboratory Medicine, Zhujiang Hospital, Southern Medical University, Guangzhou, Guangdong, China; g Department of Laboratory Medicine, The First Affiliated Hospital of Guangzhou University, Guangzhou Medical University, Guangzhou, Guangdong, China; h Department of Civil and Environmental Engineering, University of Maryland, College Park, MD, USA; i Research Institute for Food Nutrition and Human Health, Guangzhou, Guangdong, China

**Keywords:** AMR, β-lactam, plasmid replicon, antimicrobial susceptibility, ESBLs, molecular epidemiology

## Abstract

Since antimicrobial resistance, especially β-lactam resistance genes were common in clinical *Escherichia coli* strains, this study had designed and developed multiplex amplification platform for rapid and accurate detection of such resistance genes in 542 clinical *E. coli* isolates. The obtained specimens were subjected to bacteriological examination, antimicrobial susceptibility testing, and detection of β-lactamase genes and plasmid replicons. The major virulence genes were detected by 7 groups of multiplex PCR and eight groups of multiplex PCR were designed to detect 8 different plasmid replicons including *parA-parB*, iteron, *repA*, and *RNAI*. It was found that most MDR isolates were co-resistant to penicillins (AMP) and fluoroquindones (LVX, CIP) and distribution of LVX and CIP resistance was significantly higher among female than male gender. *RNAI* (AY234375) showed the highest detection rate, followed by the *iteron* (J01724) and *repA* (M26308), indicating the relatively higher carriage rate of corresponding plasmids. *Bla*_OXA_ acquired the highest carriage rate, followed by group 2 *bla*_CTX-M_ and *bla*_SHV-1_, indicating their prevalence among clinical *E. coli*. Among the β-lactamase genes, *bla*_OXA_ acquired the highest carriage rate, followed by group 2 *bla*_CTX-M_ and *bla*_SHV-1_, indicating their prevalence among clinical *E. coli*. The *RNAI* (AY234375) showed the highest detection rate, followed by the *iteron* (J01724) and *repA* (M26308), indicating the relatively higher carriage rate of the corresponding plasmids by clinical *E. coli* isolates. It is shown that the developed multiplex amplification methodology is applicable to AMR detection, and such identification of plasmid replicons and β-lactamase genes may aid in the understanding of clinical *E. coli* isolate epidemiology.

## Graphical Abstract

**Figure:**
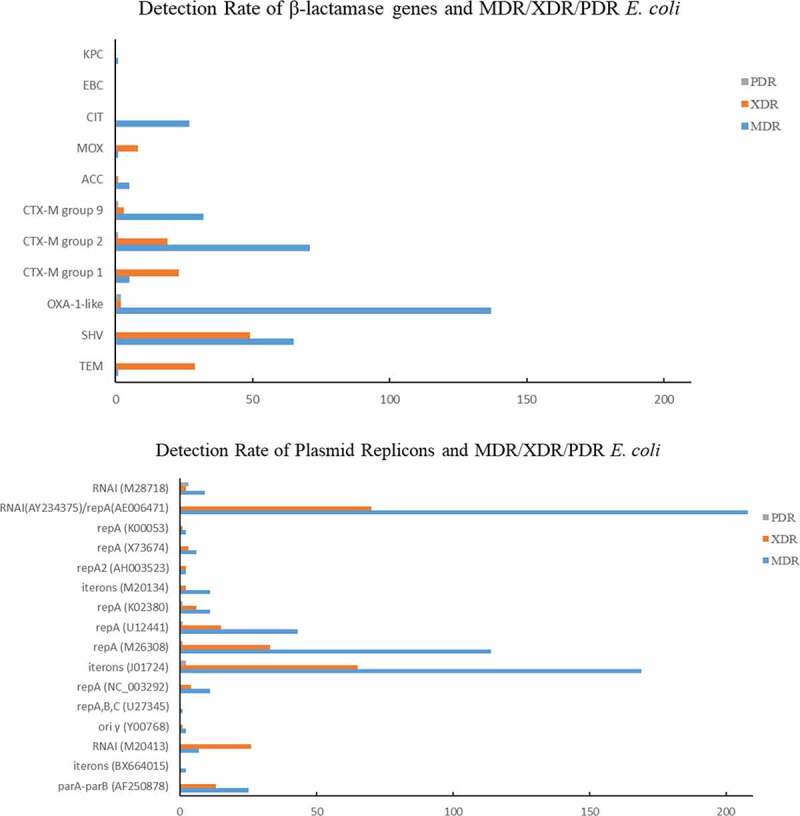

## 1. Introduction

In recent decades, antimicrobial resistance (AMR) in microorganisms has been considered to be a leading concern for human health, which has posed a significant obstacle for therapeutic treatment on various clinical infections. In 2016, the United Nations High-Level meeting had declared that progress toward achieving several of the Sustainable Development Goals (SDGs) is threatened by AMR [1]. In 2020, the World Health Organization (WHO) had announced the launch of 2 new AMR indicators in the monitoring framework of the SDGs linked to the health target 3, which monitor proportion of bloodstream infections (BSIs) due to 2 typical Super-bugs, *Escherichia coli* and Staphylococcus aureus [2]. Consequently, in this study, a strategy design based on multiplex amplification and further application for rapid detection on antimicrobial resistance and β-lactam resistance genes had been performed on a large scale of clinical *Escherichia coli* strains [3].

Bacterial factors are reported to be associated with *E. coli* pathogenesis and progression [4]. Moreover, pathogenic *E. coli* strains show great diversity in gene content, virulence factors, genomic Islands, and pathogenicity Islands [5]. Pathogenic *Escherichia coli* are capable of causing various diseases in humans, including several types of diarrhea, urinary tract infections, sepsis, and meningitis [6,7]. Due to the widely application of antibiotics, the emergence of *E. coli* isolates with multiple antibiotic-resistant phenotypes, involving coresistance to four or more unrelated families of antibiotics, has been considered a serious health concern [8,9]. A large number of genes are responsible for antibiotic resistance. As the first identified antibiotic, β-lactams have become widely applied. Introduction of β-lactams in clinical settings was quickly followed by the emergence of numerous β-lactamases [10]. Identification of β-lactamase genes is important to understand resistance epidemiology and identify resistant strains [11]. β-lactamase genes has been reported to frequently transferred by plasmids [11–13]. The spread of extended-spectrum β-lactamases (ESBLs) is an emerging global public health problem. Until the late 1990s, TEM and SHV enzymes were the most common extended-spectrum β-lactamases (ESBLs). In the late 1990s, the prevalence of both TEM and SHV decreased, whereas that of CTX-M increased, especially associated with *Escherichia coli* species. Within a few years, CTX-M ESBL-producing *E. coli* had spread across the world, involved in both nosocomial outbreaks and community-acquired infections [14].

Plasmids are capable of increasing bacterial genetic diversity, acquiring and losing genes, and can be horizontally exchanged among bacterial populations by conjugation or mobilization [15]. Plasmids contain genes essential for initiation and control of replication and accessory genes [16,17]. Replicons contribute to the replication of plasmid and represent the acquisition of plasmids.

This study collected and analyzed the 10-year antimicrobial resistance data of clinical *E. coli* isolates and aimed in identifying the distribution of β-lactamase genes and plasmid replicons, which may aid in the understanding of its epidemiology and the development of control strategies.

## 2. Material and methods

### 2.1 Bacterial strain

During 2008 to 2018, a total of 542 *E. coli* isolates were isolated from the First Affiliated Hospital of Guangzhou Medical University (FAHGMU). The large proportion of patients in FAHGMU are from Guangdong and other adjacent provinces, which can represent the epidemiology of *E. coli* in Southern China. Both inpatients and outpatients were included in this study. For the criteria of bacterial selection, strains were averagely distributed in different months, with an average of 4 to 5 strains isolated in 1 month. Also, strains were selected according to the location of medical settings and wards, as well as their antimicrobial resistance profile. The isolates datas included demographic information such as date, age, gender of patients, department of isolation and infection types.

### 2.2 Bacterial Isolation and Identification

Bacterial identification to the species level on all tested strains had been performed by standard procedures: colony morphology, Gram staining, the API commercial kit and the Vitek 2 automated system [18]. Isolation of *E. coli* was performed using standard bacteriological methods. The clinical samples were incubated in Violet Red Bile Agar (VRBA, Huankai Microbial, China) for 24 h at 37°C and those pink colored presumptive *E. coli* were sub-cultured in Eosin Methylene Blue (EMB, Huankai Microbial, China) agar to get a pure colony. Colonies with metallic green sheen were characterized micro-scopically using Gram stain and other different biochemical tests such as indole production, methyl-red test, Voges-Proskauer test, citrate utilization (IMViC) test, oxidase test, sugar fermatation, triple sugar iron [19]. Furthermore, the identified isolates were confirmed by using a species-specific set of primers targeting 16S rRNA gene of *E. coli* (27FYM: 5′- AGAGTTTGATYMTGGCTCAG-3′; 1492 R: 5′- GGTTACCTTGTTACGACTT-3′) [20].

### 2.3 Antimicrobial susceptibility testing

Antimicrobial susceptibility testing was performed by disk diffusion according to the Clinical and Laboratory Standard Institute (CLSI), and minimum inhibitory concentrations (MICs) determined through Vitek 2 automated system (Vitek AMS; bioMerieux Vitek Systems Inc., Hazelwood, MO) [17–19]. The 13 tested antimicrobial agents classified into 7 antibiotic groups: penicillins (AMP: Ampcillins), β-lactam (TZP: piperacillin-tazobactam; AMC: Amoxicillin-clavulanate), cephems (CAZ: ceftazidime; FEP: cefepime), monobactams (ATM: aztreonam), carbapenems (IPM: imipenem; MEM: meropenem), aminoglycosides (GEN: gentamicin; TOB: tobramycin; AMK: amikacin) and fluoroquinolones (LVX: levofloxacin; CIP: ciprofloxacin). In addition, the susceptibility analysis was interpreted based on the criteria on *E. coli* of CLSI (2019).

Multidrug-resistant (MDR) was defined as acquired non-susceptibility to at least one agent in more than three antimicrobial categories, and extensively drug-resistant (XDR) was defined as non-susceptibility to at least one agent in all but two or fewer antimicrobial categories [21]. Pandrug-resistant (PDR) was defined as non-susceptibility to in all antimicrobial categories listed.

### 2.4 Detection of β-lactamase genes

The major β-lactamase genes were detected by seven groups of multiplex PCR [22,23]. Three sets of primers were used in group 1 for the amplication of *bla*_TEM_ (*bla*_TEM-1_ and *bla*_TEM-2_), *bla*_SHV-1_, and *bla*_OXA_ (*bla*_OXA-1_, *bla*_OXA-4_ and *bla*_OXA-30_) genes, respectively. Group 2 and group 3 mainly amplified *bla*_CTX-M_, including group 1 *bla*_CTX-M_ (*bla*_CTX-M-1_, *bla*_CTX-M-3_ and *bla*_CTX-M-15_), group 2 *bla*_CTX-M_ (*bla*_CTX-M-2_) and group 9 *bla*_CTX-M_ (*bla*_CTX-M9-_ and *bla*_CTX-M-14_), and group 8/25 *bla*_CTX-M_ (*bla*_CTX-M-8_, *bla*_CTX-M-25_, *bla*_CTX-M-26_ and *bla*_CTX-M-39_ to *bla*_CTX-M-41_). Five sets of primers were used in group 4 to detect *bla*_AAC_ (*bla*_AAC-1_ and *bla*_AAC-2_), *bla*_FOX_ (*bla*_FOX-1_ to *bla*_FOX-5_), *bla*_MOX_ (*bla*_MOX-1_, *bla*_MOX-2_, *bla*_CMY-1_, *bla*_CMY-8_ to *bla*_CMY-11_ and *bla*_CMY-19_), *bla*_DHA_ (*bla*_DHA-1_ and *bla*_DHA-2_), *bla*_CIT_ (*bla*_LAT-1_ to *bla*_LAT-3_, *bla*_BIL-1_, *bla*_CMY-2_ to *bla*_CMY-7_, *bla*_CMY-12_ to *bla*_CMY-18_ and *bla*_CMY-21_ to *bla*_CMY-23_) and *bla*_EBC_ (*bla*_ATC-1_ and *bla*_MIR-1_). *Bla*_VEB_ (*bla*_VEB-1_ to *bla*_VEB-6_), *bla*_PER_ (*bla*_PER-1_ and *bla*_PER-3_) and *bla*_GES_ (*bla*_GES-1_ to *bla*_GES-9_ and *bla*_GES-11_) genes were detected by group 5. *Bla*_GES_ (*bla*_GES-1_ to *bla*_GES-9_ and *bla*_GES-11_) and *bla*_OXA-18_ were amplified by group 6, while *bla*_IMP_, *bla*_VIM_ (*bla*_VIM-1_ and *bla*_VIM-2_) and *bla*_KPC_ (*bla*_KPC-1_ to *bla*_KPC-5_) were detected by group 7. Annealing temperature at 60°C was used for group 1–5, while 57°C and 55°C were selected for group 6 and 7, respectively. One min was chosen for extention time since all the PCR products were shorter than 1 kb. All PCR products were run on 2% agarose gel. To further avoid false-positive results from multiplex PCR, all positive samples were further subjected to separate PCR confirmation using single pairs of primers from the corresponding multiplex PCR. For samples yielding positive results from single PCR reaction, Sanger sequencing was further performed to ensure accuracy. All experiments were performed in triplicate.

### 2.5 Detection of plasmid replicons

Eight groups of multiplex PCR were designed to detect 8 different plasmid replicons [22,23]. Group 1 targeted on *parA-parB* (AF250878), *iteron* (BX664015), and *RNAI* (M20413). The target genes in group 2 were *oriγ* (Y00768), *repABC* (U27345), and *repA* (NC_003292). One iteron (J01724) and two *repA* genes (M26308 and U12441) were selected to be detected in group 3. Similarly, iteron (M20134), *repA* (K02380), and *repA2* (AH003523) were detected in group 4. Three *repA* genes (X73674, K00053, and AE006471) were amplified in group 5. In group 6–8, three different *RNAI* (AY234375, M93063, and M28718) were detected, respectively. For the multiplex PCR groups except for group 6, annealing temperature at 60°C and extention for 1 min were used. In group 6, 52°C was selected for annealing temperature. All PCR products were run on 2% agarose gel.

### 2.6 Statistical analysis

In this study, the susceptibility of *E. coli* was defined as resistant (resistant and intermediate resistant) and susceptible in this study. The antimicrobial susceptibility test results were collected and managed in WHONET (version 5.6). Chi-square test or Fisher’s exact test were used to analyze the difference of proportions, if appropriate. A value of *p* < 0.05 was defined as statistically significant.

## 3. Results

The obtained *E. coli* isolates were subjected to bacteriological examination, antimicrobial susceptibility testing, detection of β-lactamase genes and plasmid replicons. The identification of plasmid replicons and β-lactamase genes, especially MDR, XDR, and PDR strains, may aid in the understanding of clinical *E. coli* isolates epidemiology.

### 3.1 Phenotypic characteristics of the recovered isolates

The colonies of the recoverd *E. coli* isolates showed bright pink colonies on VRBA agar plates and showed a distinctive metallic green sheen on EMB agar. All *E. coli* isolates were lactose fermenting colonies, methyl red, pidole-positive, catalase, oxidase-negative, urea hydrolysisi, critrate utilization, Voges-Proskauer, and did produce H_2_S in biochemical tests. Moreover, all isolates PCR amplicons were compared in the GenBank database using the BLAST program and the result indicated that these strains belonged to *Escherichia coli*.

### 3.2 *Characteristics of antimicrobial resistance of* E. coli

*E. coli* isolates collected from 7 different specimen sources during 10-year period were compared antimicrobial resistance in this study (Table 1). Females were the most affected group of patients (286 samples, 58.4%) as compared to males (181 samples, 37.0%). Maximum number of *E. coli* were isolated in the age group of 41–70 (271 samples, 55.3%). Urine was the most predominant specimen source of *E. coli* (46.9%), followed by blood (24.7%) and sputum (12.2%). Other remaining sources included secretion, pleural fluid, pus and wound, only making up a small proportion.

**Table 1.** Characteristics of specimen and drug resistance profiles of *E. coli.*

		Total/MDR isolate numbers (%)									
		2008	2009	2010	2011	2012	2013	2014	2015	2016	2017(51)
Total		48/36(75.0)	47/36(76.6)	49/39(79.6)	47/39(80.9)	57/50(87.7)	36/24(66.7)	48/40(83.3)	33/25(83.3)	74/56(75.7)	51/33(64.7)
Gender											
Male		21(43.8) /16(44.4)	16(34.0)/13(76.6)	22(44.9)/19(48.7)	13(27.7)/12(31.6)	24(42.1)/21(42.0)	16(44.4)/10(41.7)	16(33.3)/12(30.0)	16(48.5)/11(44.0)	31(41.9)/24(42.9)	6(11.8)/11(33.3)
Female		27(56.3)/20(55.6)	31(66.0)/23(63.9)	27(55.1)/20(15.3)	34(72.3)/26(68.4)	33(57.9)/29(58.0)	20(55.6)/14(58.3)	32(66.7)/28(70.0)	17(51.5)/14(56.0)	43(58.1)/32(57.1)	22(43.1)/22(66.7)
Age											
≤40		7(14.6)/5(44.4)	9(19.1)/8(22.2)	11(22.4)/9(23.1)	7(14.9)/12(18.4)	3(5.3)/3(6.0)	4(11.1)/0(0.0)	4(8.3)/4(10.0)	4(12.1)/4(16.0)	23(31.1)/10(17.9)	9(17.6)/5(15.2)
41–55		13(27.1)/10(27.8)	17(36.2)/14(38.9)	14(28.6)/12(30.8)	10(21.3)/8(21.2)	17(29.8)/13(26.0)	7(19.4)/6(25.0)	16(33.3)/11(27.5)	6(18.2)/5(20.0)	14(18.9)/12(21.4)	16(31.4)/9(27.3)
56–70		12(25.0)/7(19.4)	11(23.4)/8(22.2)	11(22.4)/9(23.1)	13(27.7)/9(23.7)	20(35.1)/17(34.0)	10(27.8)/6(25.0)	14(29.2)/13(32.5)	13(39.4)/13.32.5)	24(32.4)/21(37.5)	13(25.5)/9(27.3)
71–85		14(29.2)/12(33.3)	10(21.3)/6(16.7)	12(24.5)/8(20.5)	12(25.5)/9(23.7)	12(21.1)/10(20.0)	14(38.9)/10(41.7)	12(25.0)/10(25.0)	10(30.3)/10(25.0)	10(13.5)/10(17.9)	11(21.6)/9(27.3)
>86		2(4.2)/2(5.6)	0(0.0)/0(0.0)	1(2.0)/1(2.6)	3(6.4)/3(7.9)	4(7.0)/4(8.0)	1(2.8)/1(4.2)	2(4.2)/2(5.0)	0(0.0)/0(0.0)	3(4.1)/3(5.4)	1(2.0)/2(3.0)
Specimen											
Sputum		6(12.5)/6(16.7)	5(10.6)/4(11.1)	7(14.3)/5(12.8)	9(19.1)/8(21.1)	5(8.8)/4(8.0)	0(0.0)/0(0.0)	0(0.0)/0(0.0)	3(9.1)/0(0.0)	20(27.0)/12(21.4)	8(19.6)/9(21.2)
Urine		31(64.6)/24(66.7)	30(63.8)/26(72.2)	33(67.3)/28(71.8)	30(63.8)/24(63.2)	35(61.4)/30(60.0)	0(0.0)/0(0.0)	1(2.1)/0(0.0)	9(27.3)/6(24.0)	39(52.7)/33(58.9)	40(78.4)/24(72.7)
Sterile body fluids		2(4.2)/2(5.6)	2(4.3)/1(2.8)	2(4.1)/0(0.0)	5(10.6)/4(10.5)	7(12.3)/6(12.0)	0(0.0)/0(0.0)	1(7.0)/1(2.5)	0(0.0)/0(0.0)	4(5.4)/3(5.4)	1(0.2)/1(3.0)
Blood		4(8.3)/2(5.6)	5(10.6)/4(11.1)	4(8.2)/3(7.7)	0(0.0)/0(0.0)	4(7.0)/4(8.0)	36(100.0)/24(100.0)	46(95.8)/37(97.5)	21(63.6)/17(68.0)	1(1.4)/1(1.8)	0(0.0)/0(0.0)
Pus		2(4.2)0(0.0)	4(8.5)/0(0.0)	1(2.0)/1(2.6)	1(2.1)/1(2.6)	2(3.5)/2(4.0)	0(0.0)/0(0.0)	0(0.0)/0(0.0)	0(0.0)/0(0.0)	2(2.8)/2(4.6)	0(0.0)/0(0.0)
Wound		1(2.1)/0(0.0)	0(0.0)/0(0.0)	0(0.0)/0(0.0)	0(0.0)/0(0.0)	1(1.8)/1(2.0)	0(0.0)/0(0.0)	0(0.0)/0(0.0)	0(0.0)/0(0.0)	2(2.7)/1(1.8)	0(0.0)/0(0.0)
Others		3(6.3)/2(5.6)	1(2.1)/1(5.6)	2(4.1)/2(5.1)	2(4.3)/1(2.6)	3(5.3)/3(6.0)	0(0.0)/0(0.0)	0(0.0)/0(0.0)	0(0.0)/0(0.0)	6(8.1)/4(7.1)	2(0.4)/0(0.0)
Antimicrobial resistance (N)											
Penicillins	AMP	38(79.2)/34(94.4)	38(80.9)/35(97.2)	42(85.7)/38(97.4)	41(87.2)/37(94.8)	52(91.2)/50(100)	28(77.8)/24(100)	39(81.3)/37(92.5)	26(78.8)/24(96.0)	64(86.5)/55(98.2)	30(58.8)/28(84.8)
β-lactam	TZP	1(2.1)/1(2.8)	2(4.3)/2(55.6)	2(4.1)/2(5.1)	1(2.1)/1(2.6)	2(3.5)/2(4.0)	2(5.6)/2(8.3)	1(2.1)/1(2.5)	1(3.0)/1(4.0)	9(12.2)/9(16.1)	9(17.6)/9(27.3)
	AMC	NT ^b^	NT	NT	NT	NT	6(16.7)/6(25.0)	25(52.1)/25(62.5)	12(36.4)/12(48.0)	27(36.5)/26(46.4)	22(43.1)/18(54.5)
Cephems	CAZ	11(22.9)/11(30.6)	8(17.0)/8(22.2)	5(10.2)/5(12.8)	12(25.5)/12(30.8)	17(29.8)/17(34.0)	7(19.4)/7(29.2)	27(56.3)/27(67.5)	14(42.4)/14(56.0)	34(45.9)/33(58.9)	8(15.7)/8(24.2)
	FEP	10(20.8)/10(27.8)	21(44.7)/20(55.6)	11(22.4)/11(28.2)	15(31.9)/15(38.5)	20(35.1)/20(40.0)	10(27.8)/10(41.7)	17(35.4)/16(40.0)	8(24.2)/8(32.0)	23(31.1)/19(33.9)	19(37.3)/16(48.5)
Monobactams	ATM	16(33.3)/16(44.4)	15(31.9)/15(41.7)	18(36.7)/18(46.2)	20(42.6)/20(51.3)	27(47.4)/27(54.0)	13(36.1)/13(54.2)	21(43.8)/21(52.5)	13(39.4)/12(48.0)	30(40.5)/30(53.6)	16(31.4)/15(45.5)
Carbapenems	IPM	1(2.1)/1(2.8)	2(4.3)/2(55.6)	0(0.0)/0(0.0)	0(0.0)/0(0.0)	1(1.8)/1(2.0)	1(2.8)/1(4.2)	0(0.0)/0(0.0)	0(0.0)/0(0.0)	5(6.8)/5(8.9)	2(3.9)/2(6.1)
	MEM	3(6.3)/0(0.0)	1(2.1)/1(2.7)	3(6.1)/1(2.6)	0(0.0)/0(0.0)	0(0.0)/0(0.0)	1(2.8)/1(4.2)	0(0.0)/0(0.0)	0(0.0)/0(0.0)	4(5.4)/4(7.1)	2(3.9)/2(6.1)
Aminoglycosides	GEN	29(60.4)/29(80.6)	22(46.8)/22(61.1)	22(44.9)/22(56.4)	35(74.5)/34(87.2)	33(57.9)/33(66.0)	12(33.3)/12(50.0)	24(50.0)/24(60.0)	15(45.5)/15(60.0)	28(37.8)/26(46.4)	19(37.3)/18(54.5)
	TOB	27(56.3)/27(75.0)	20(42.6)/20(55.6)	16(32.7)/16(41.0)	30(63.8)/30(78.9)	29(50.9)/29(58.0)	12(33.3)/12(50.0)	28(58.3)/27(67.5)	14(42.4)/14(56.0)	28(37.8)/28(50.0)	19(37.3)/19(57.6)
	AMK	1(2.1)/1(2.8)	7(14.9)/2(5.6)	2(4.1)/2(5.1)	2(4.3)/1(2.6)	6(10.5)/6(12.0)	1(2.8)/1(4.2)	3(6.3)/3(7.5)	1(3.0)/1(4.0)	3(4.1)/3(5.4)	3(5.9)/3(9.1)
Fluoroquindones	LVX	41(85.4)/36(100)	41(87.2)/36(100)	40(81.6)/39(100)	40(85.1)/39(100)	51(89.5)/48(96.0)	30(83.3)/23(95.8)	44(91.7)/40(100)	28(84.8)/24(96.0)	59(79.7)/52(92.9)	46(90.2)/33(100)
	CIP	39(81.3)/34(94.4)	38(80.9)/35 (97.2)	35(71.4)/35(89.7)	36(76.6)/36(92.3)	46(80.7)/45(90.0)	23(63.9)/22(91.7)	34(70.8)/33(82.5)	21(63.6)/18(72.0)	50(67.6)/47(83.9)	28(54.9)/26(78.8)

AMP: Ampcillins; TZP: Piperacillin-tazobactam; AMC: Amoxicillin-clavulanate; CAZ: Ceftazidime; FEP: Cefepime; ATM: Aztreonam; IPM: Imipenem; MEM: Imipenem; GEN: Gentamicin; TOB: Tobramycin; AMK: Amikacin; LVX: Levofloxacin; CIP: Ciprofloxacin.

MDR: non-susceptibility to ≥1 agent in ≥3 antimicrobial categories; XDR: non-susceptibility to ≥1 agent in all but ≤2 antimicrobial categories; PDR: non-susceptibility to in all antimicrobial categories listed.

^a^N (%), ^b^ NT: No Tested

**Figure 1.**
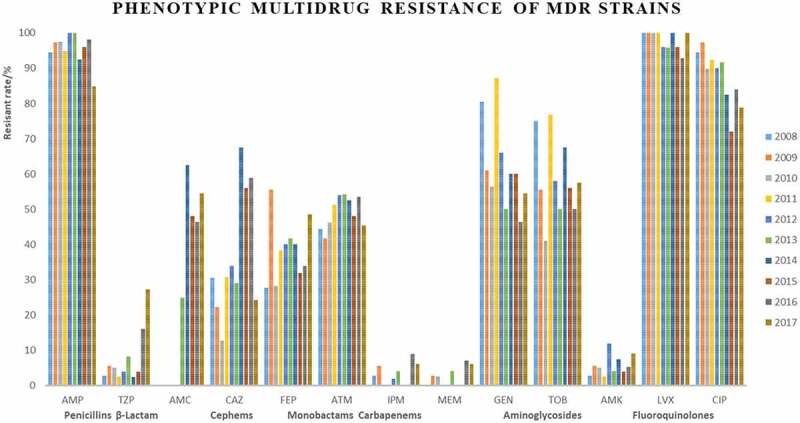
Phenotypic Multidrug Resistance of MDR Strains.

The resistance rates of penicillins (AMP) and fluoroquindones (LVX, CIP) remained high during the study period (vary between 58.8% and 91.2%, 79.7% and 90.2%, 54.9% and 81.3% over the study period, respectively; Figure 1). Monobactams (ATM) and aminoglycosides (GEN, TOB) kept fluctant (between 31.9% and 74.5%). Meanwhile, increased rates in β-lactam use for clinical treatment accompanied these increasing resistance rates, the resistance rate of β-lactam (TZP, AMC, 3.5% to 40.7%) and cephems (CAZ, FEP, 16.7% to 40.3%) increased during 10-year period.

### 3.3 *MDR, XDR and PDR profiles in* E. coli

Approximately 92.9% of isolates were resistant to at least one antimicrobial agent (Table 1). Among them, 76.9% 27.9%, 1.4% were MDR, XDR, and PDR, respectively (Table 2). The results showed that many MDR isolates were co-resistant to penicillins (AMP) and fluoroquindones (LVX, CIP) and distribution of LVX and CIP resistance was significantly higher among female than male gender. These results were not exactly consistent with previous research [24,25], its reasons may be due to the geographical difference of *E. coli*.

**Table 2.** Characteristics of antimicrobial susceptibility testing profiles of *E. coli.*

Year (Total)		2008(48)	2009(47)	2010(49)	2011(47)	2012(57)	2013(36)	2014(48)	2015(33)	2016(74)	2017(51)
MDR		36(75.0) ^a^	36(76.6)	39(79.6)	39(80.9)	50(87.7)	24(66.7)	40(83.3)	25(83.3)	56(75.7)	33(64.7)
XDR		9(18.8)	13(27.7)	10(20.4)	13(27.7)	17(29.8)	9(25.0)	19(39.6)	11(33.3)	24(32.4)	12(23.5)
PDR		1(2.8)	1(2.1)	0(0.0)	0(0.0)	1(1.8)	1(2.8)	0(0.0)	0(0.0)	2(2.7)	1(2.0)
Antimicrobial resistance of		MDR/XDR/PDR									
Penicillins	AMP	34(94.4)/9(100)/1(100)	35(97.2)/12(92.3)/1(100)	38(97.4)/10(100)/0(0.0)	37(94.8)/13(0.0)/0(0.0)	50(100)/17(100)/1(100)	24(100)/9(100)/1(100)	37(92.5)/19(100)/0(0.0) /0(0.0)	24(96.0)/11(100)/0(0.0)	55(98.2)/24(100)/2(100)	28(84.8)/11(91.7)/1(100)
β-lactam	TZP	1(2.8)/0(0.0)/1(100)	2(55.6)/1(7.7)/1(100)	2(5.1)/10(100)/0(0.0)	1(2.6)/0(0.0)/0(0.0)	2(4.0)/1(5.9)/1(100)	2(8.3)/1(11.1)/1(100)	1(2.5)/1(5.3)/0(0.0)	1(4.0)/1(9.1)/0(0.0)	9(16.1)/5(20.8)/2(100)	9(27.3)/6(50.0)/1(100)
	AMC	NT ^b^	NT	NT	NT	NT	6(25.0)/2(22.2)/0(0.0)	25(62.5)/14(73.9)/0(0.0)	12(48.0)/6(5.5)/0(0.0)	26(46.4)/15(62.5)/2(100)	18(54.5)/10(83.3)/1(100)
Cephems	CAZ	11(30.6)/6(66.7)/1(100)	8(22.2)/5(38.5)/1(100)	5(12.8)/5(50.0)/0(0.0)	12(30.8)/10(76.9)/0(0.0)	17(34.0)/14(82.4)/1(100)	7(29.2)/6(66.7)/1(100)	27(67.5)/19(100)/0(0.0)	14(56.0)/11(100)/0(0.0)	33(58.9)/23(95.8)/2(100)	8(24.2)/7(58.3)/0(0.0)
	FEP	10(27.8)//7(77.8)/1(100)	20(55.6)/13(100)/1(100)	11(28.2)/9(90.0)/0(0.0)	15(38.5)/12(92.3)/0(0.0)	20(40.0)/15(88.2)/1(100)	10(41.7)/8(88.9)/1(100)	16(40.0)15(78.9)/0(0.0)	8(32.0)/8(72.7)/0(0.0)	19(33.9)/14(58.3)/2(100)	16(48.5)/10(83.3)/1(100)
Monobactams	ATM	16(44.4)/9(100)/1(100)	15(41.7)/12(92.3)/1(100)	18(46.2)/9(90.0)/0(0.0)	20(51.3)/13(100)/0(0.0)	27(54.0)/17(100)/1(100)	13(54.2)/9(100)/1(100)	21(52.5)17(89.5)/0(0.0)	12(48.0)/11(100)/0(0.0)	30(53.6)/22(91.7)/2(100)	15(45.5)/9(75.0)/1(100)
Carbapenems	IPM	1(2.8)/0(0.0)/1(100)	2(55.6)/1(7.7)/1(100)	0(0.0)/0(0.0)/0(0.0)	0(0.0)/0(0.0)/0(0.0)	1(2.0)/1(100)/0(0.0)	1(4.2)/0(0.0)/1(100)	0(0.0)/0(0.0)/0(0.0)	0(0.0)/0(0.0)/0(0.0)	5(8.9)/1(4.2)/2(100)	2(6.1)/1(8.3)/1(100)
	MEM	0(0.0)/0(0.0)/0(0.0)	1(2.7)/1(7.7)/0(0.0)	1(2.6)/1(10.0)/0(0.0)	0(0.0)/0(0.0)/0(0.0)	0(0.0)/0(0.0)/0(0.0)	1(4.2)/0(0.0)/1(100)	0(0.0)/0(0.0)/0(0.0)	0(0.0)/0(0.0)/0(0.0)	4(7.1)/2(8.3)/2(100)	2(6.1)/1(8.3)/1(100)
Aminoglycosides	GEN	29(80.6)/8(88.9)/1(100)	22(61.1)/11(84.6)/1(100)	22(56.4)/9(90.0)/0(0.0)	34(87.2)/13(100)/0(0.0)	33(66.0)/13(76.5)/1(100)	12(50.0)/6(66.7)/1(100)	24(60.0)/15(78.9)/0(0.0)	15(60.0)/8(72.7)/0(0.0)	26(46.4)/16(6.7)/1(50.0)	18(54.5)/10(83.3)/0(0.0)
	TOB	27(75.0)/9(100)/0(0.0)	20(55.6)/12(92.3)/1(100)	16(41.0)/8(80.0)/0(0.0)	30(78.9)/12(92.3)/0(0.0)	29(58.0)/12(70.6)/1(100)	12(50.0)6(66.7)/1(100)	27(67.5)/15(78.9)/0(0.0)	14(56.0)/8(72.7)/0(0.0)	28(50.0)/15(62.5)/2(100)	19(57.6)10(83.3)/1(100)
	AMK	1(2.8)/0(0.0)/0(0.0)	2(5.6)/1(7.7)/1(100)	2(5.1)/2(20.0)/0(0.0)	1(2.6)/1(7.7)/0(0.0)	6(12.0)/3(17.6)/0(0.0)	1(4.2)/1(11.1)/0(0.0)	3(7.5)/2(10.5)/0(0.0)	1(4.0)/1(9.1)/0(0.0)	3(5.4)/1(4.2)/0(0.0)	3(9.1)/3(25.0)/0(0.0)
Fluoroquindones	LVX	36(100)/9(100)/1(100)	36(100)/13(100)/1(100)	39(100)/10(100)/0(0.0)	39(100)/13(100)/0(0.0)	48(96.0)/17(100)/1(100)	23(95.8)/9(100)/1(100)	40(100)/19(100)/0(0.0)	24(96.0)/11(100)/0(0.0)	52(92.9)/24(100)/2(100)	33(100)/12(100)/1(100)
	CIP	34(94.4)/9(100)/1(100)	35(97.2)/13(100)/1(100)	35(89.7)/10(100)/0(0.0)	36(92.3)/12(92.3)/0(0.0)	45(90.0)/17(100)/1(100)	22(91.7)/9(100)/1(100)	33(82.5)/17(89.5)/0(0.0)	18(72.0)/10(91.0)/0(0.0)	47(83.9)/23(95.8)/2(100)	26(78.8)/11(91.7)/1(100)

AMP: Ampcillins; TZP: Piperacillin-tazobactam; AMC: Amoxicillin-clavulanate; CAZ: Ceftazidime; FEP: Cefepime; ATM: Aztreonam; IPM: Imipenem; MEM: Imipenem; GEN: Gentamicin; TOB: Tobramycin; AMK: Amikacin; LVX: Levofloxacin; CIP: Ciprofloxacin.

MDR: non-susceptibility to ≥1 agent in ≥3 antimicrobial categories; XDR: non-susceptibility to ≥1 agent in all but ≤2 antimicrobial categories; PDR: non-susceptibility to in all antimicrobial categories listed.

^a^N (%), ^b^ NT: No Tested

**Figure 2.**
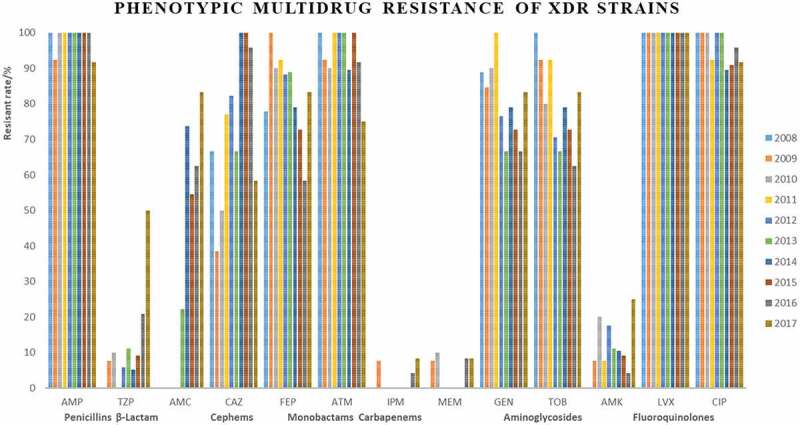
Phenotypic Multidrug Resistane of XDR Strains.

**Figure 3.**
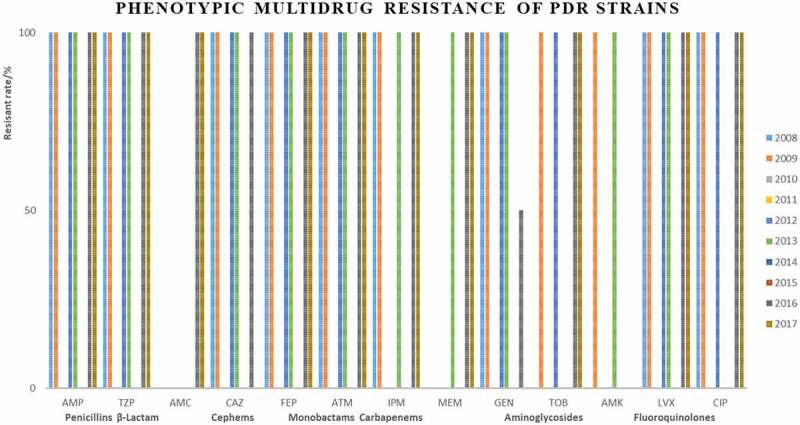
Phenotypic Multidrug Resistane of PDR Strains.

Strains Most of XDR strains were resistant to penicillins, cephems, monobactams, and fluoroquinolones (Figure 2). Only 7 strains were identified as PDR strains, isolated in 2008, 2009, 2012, 2013, 2016, and 2017 (Table 2, Figure 3).

### 3.4 Distribution of β-lactamase genes

According to characteristics of antimicrobial resistance to β-lactam (TZP, AMC), the resistance rate increased during the study period (from 3.5% to 40.8%). Among all β-lactamase genes, *bla*_OXA-1_ acquired the highest carriage rate (195/542, 35.98%), followed by group 2 *bla*_CTX-M_ (90/542, 16.61%), *bla*_SHV-1_ (78/542, 14.39%) and group 9 *bla*_CTX-M_ (39/542, 7.20%), indicating their prevalence among clinical *E. coli* in South China (Table 3). The carriage rate of *bla*_OXA-1_ was high (40.82–62.50%) from 2008 to 2012, 2015, and 2018, but decreased (4.08–5.56%) in 2013–2014 and 2016–2017. The carriage rate of group 2 *bla*_CTX-M_ and *bla*_SHV-1_ was fluctuant.

**Table 3.** Distribution of β-lactamase genes and plasmid replicons of *E. coli.*

Genes	Primer sequence (5’-3’)	2008 (48)	2009 (47)	2010 (49)	2011 (48)	2012 (57)	2013 (36)	2014 (49)	2015 (36)	2016 (72)	2017 (51)	2018 (49)	Total (542)
	β-lactamase (BL)												
TEM	F: CATTTCCGTGTCGCCCTTATTC	0(0.0)	0(0.0)	0(0.0)	1(2.1)	0(0.0)	0(0.0)	0(0.0)	0(0.0)	0(0.0)	0(0.0)	0(0.0)	1(0.2)
	R: CGTTCATCCATAGTTGCCTGAC												
SHV	F: AGCCGCTTGAGCAAATTAAAC	2(4.2)	0(0.0)	5(10.2)	3(6.3)	1(1.8)	8(22.2)	13(26.5)	0(0.0)	20(27.8)	25(49.0)	1(2.0)	78(14.4)
	R: ATCCCGCAGATAAATCACCAC												
OXA-1	F: GGCACCAGATTCAACTTTCAAG	30(62.5)	28(57.4)	20(40.8)	23(47.9)	32(56.1)	2(5.6)	2(4.1)	16(44.4)	5(6.9)	4(7.8)	34(69.4)	195(36.0)
	R: GACCCCAAGTTTCCTGTAAGTG												
CTX-1	F: TTAGGAARTGTGCCGCTGYA ^b^	2(4.2)	4(8.5)	0(0.0)	0(0.0)	0(0.0)	0(0.0)	0(0.0)	0(0.0)	0(0.0)	0(0.0)	0(0.0)	6(1.1)
	R: CGATATCGTTGGTGGTRCCAT ^b^												
CTX-2	F: CGTTAACGGCACGATGAC	19(39.6)	20(42.6)	4(8.2)	5(10.4)	19(33.3)	0(0.0)	0(0.0)	2(5.6)	11(15.3)	5(9.8)	5(10.2)	90(16.6)
	R: CGATATCGTTGGTGGTRCCAT ^b^												
CTX-9	F: TCAAGCCTGCCGATCTGGT	4(8.3)	3(6.4)	4(8.2)	5(10.4)	12(21.1)	0(0.0)	4(8.2)	0(0.0)	1(2.4)	0(0.0)	6(12.2)	39(7.2)
	R: TGATTCTCGCCGCTGAAG												
CTX-8/25	F: AACRCRCAGACGCTCTAC ^b^	0(0.0)	0(0.0)	0(0.0)	0(0.0)	0(0.0)	0(0.0)	0(0.0)	0(0.0)	0(0.0)	0(0.0)	0(0.0)	0(0.0)
	R: TCGAGCCGGAASGTGTYAT ^b^												
ACC	F: CACCTCCAGCGACTTGTTAC	0(0.0)	0(0.0)	3(6.1)	2(4.2)	1(1.8)	0(0.0)	0(0.0)	0(0.0)	0(0.0)	0(0.0)	0(0.0)	6(1.1)
	R: GTTAGCCAGCATCACGATCC												
FOX	F: CTACAGTGCGGGTGGTTT	0(0.0)	0(0.0)	0(0.0)	0(0.0)	0(0.0)	0(0.0)	0(0.0)	0(0.0)	0(0.0)	0(0.0)	0(0.0)	0(0.0)
	R: CTATTTGCGGCCAGGTGA												
MOX	F: GCAACAACGACAATCCATCCT	0(0.0)	0(0.0)	0(0.0)	0(0.0)	0(0.0)	0(0.0)	0(0.0)	0(0.0)	0(0.0)	0(0.0)	0(0.0)	2(0.37)
	R: GGGATAGGCGTAACTCTCCCAA												
DHA	F: TGATGGCACAGCAGGATATTC	0(0.0)	0(0.0)	0(0.0)	0(0.0)	0(0.0)	0(0.0)	0(0.0)	0(0.0)	0(0.0)	0(0.0)	0(0.0)	0(0.0)
	R: GCTTTGACTCTTTCGGTATTCG												
CIT	F: CGAAGAGGCAATGACCAGAC	0(0.0)	0(0.0)	21(42.9)	10(20.8)	0(0.0)	0(0.0)	0(0.0)	0(0.0)	1(1.4)	0(0.0)	0(0.0)	32(5.9)
	R: ACGGACAGGGTTAGGATAGY ^b^												
EBC	F: CGGTAAAGCCGATGTTGCG	0(0.0)	0(0.0)	0(0.0)	0(0.0)	0(0.0)	0(0.0)	0(0.0)	0(0.0)	0(0.0)	0(0.0)	1(2.0)	1(0.2)
	R: AGCCTAACCCCTGATACA												
GES	F: AGTCGGCTAGACCGGAAAG	0(0.0)	0(0.0)	0(0.0)	0(0.0)	0(0.0)	0(0.0)	0(0.0)	0(0.0)	0(0.0)	0(0.0)	0(0.0)	0(0.0)
	R: TTTGTCCGTGCTCAGGAT												
PER	F: GCTCCGATAATGAAAGCGT	0(0.0)	0(0.0)	0(0.0)	0(0.0)	0(0.0)	0(0.0)	0(0.0)	0(0.0)	0(0.0)	0(0.0)	0(0.0)	0(0.0)
	R: TTCGGCTTGACTCGGCTGA												
VEB	F: CATTTCCCGATGCAAAGCGT	0(0.0)	0(0.0)	0(0.0)	0(0.0)	0(0.0)	0(0.0)	0(0.0)	0(0.0)	0(0.0)	0(0.0)	0(0.0)	0(0.0)
	R: CGAAGTTTCTTTGGACTCTG												
OXA-48	F: GCTTGATCGCCCTCGATT	0(0.0)	0(0.0)	0(0.0)	0(0.0)	0(0.0)	0(0.0)	0(0.0)	0(0.0)	0(0.0)	0(0.0)	0(0.0)	0(0.0)
	R: GATTTGCTCCGTGGCCGAAA												
IMP	F: TTGACACTCCATTTACDG ^b^	0(0.0)	0(0.0)	0(0.0)	0(0.0)	0(0.0)	0(0.0)	0(0.0)	0(0.0)	0(0.0)	0(0.0)	0(0.0)	0(0.0)
	R: GATYGAGAATTAAGCCACYCT ^b^												
VIM	F: GATGGTGTTTGGTCGCATA	0(0.0)	0(0.0)	0(0.0)	0(0.0)	0(0.0)	0(0.0)	0(0.0)	0(0.0)	0(0.0)	0(0.0)	0(0.0)	0(0.0)
	R: CGAATGCGCAGCACCAG												
KPC	F: CATTCAAGGGCTTTCTTGCTGC	0(0.0)	0(0.0)	1(2.0)	0(0.0)	0(0.0)	0(0.0)	0(0.0)	0(0.0)	0(0.0)	0(0.0)	0(0.0)	1(0.2)
	R: ACGACGGCATAGTCATTTGC												
	PlasmidsReplicons(PR)												
parA-parB (AF250878)	F: GGAGCGATGGATTACTTCAGTAC	1(2.1)	0(0.0)	0(0.0)	0(0.0)	5(8.8)	0(0.0)	0(0.0)	0(0.0)	11(15.3)	12(23.5)	2(4.1)	31(5.7)
	R: TGCCGTTTCACCTCGTGAGTA												
Iterons (BX664015)	F: TTTCTCCTGAGTCACCTGTTAACAC	0(0.0)	1(2.1)	1(2.0)	0(0.0)	0(0.0)	0(0.0)	0(0.0)	0(0.0)	1(1.4)	3(5.9)	0(0.0)	6(1.1)
	R: GGCTCACTACCGTTGTCATCCT												
RNAI (M20413)	F: CGAAAGCCGGACGGCAGAA	4(8.3)	5(10.6)	8(16.3)	8(16.7)	6(10.5)	0(0.0)	5(10.2)	6(16.7)	10(13.9)	6(11.8)	3(6.1)	61(11.3)
	R: TCGTCGTTCCGCCAAGTTCGT												
ori γ (Y00768)	F: AACCTTAGAGGCTATTTAAGTTGCTGAT	0(0.0)	0(0.0)	0(0.0)	0(0.0)	2(3.5)	0(0.0)	0(0.0)	0(0.0)	0(0.0)	0(0.0)	11(22.4)	13(2.4)
	R: TGAGAGTCAATTTTTATCTCATGTTTTAGC												
repA,B,C (U27345)	F: GGATGAAAACTATCAGCATCTGAAG	0(0.0)	1(2.1)	0(0.0)	1(2.1)	0(0.0)	0(0.0)	0(0.0)	0(0.0)	0(0.0)	0(0.0)	3(6.1)	5(0.9)
	R: CTGCAGGGGCGATTCTTTAGG												
repA (NC_003292)	F: GTCTAACGAGCTTACCGAAG	2(4.2)	0(0.0)	4(8.2)	1(2.1)	0(0.0)	0(0.0)	0(0.0)	4(11.1)	2(2.8)	4(7.8)	7(14.3)	24(4.4)
	R: GTTTCAACTCTGCCAAGTTC												
Iterons (J01724)	F: CCATGCTGGTTCTAGAGAAGGTG	15(31.3)	18(38.3)	18(36.7)	18(37.5)	27(47.4)	21(58.3)	37(75.5)	24(66.7)	30(41.7)	4(7.8)	21(42.9)	233(43.0)
	R: GTATATCCTTACTGGCTTCCGCAG												
repA (M26308)	F: GGAGTTCTGACACACGATTTTCTG	26(54.2)	36(76.6)	37(75.5)	28(58.3)	0(0.0)	0(0.0)	0(0.0)	0(0.0)	0(0.0)	0(0.0)	0(0.0)	135(24.9)
	R: CTCCCGTCGCTTCAGGGCATT												
repA (U12441)	F: CCTAAGAACAACAAAGCCCCCG	0(0.0)	0(0.0)	0(0.0)	0(0.0)	13(22.8)	0(0.0)	11(22.5)	11(30.6)	11(15.3)	0(0.0)	0(0.0)	46(8.5)
	R: GGTGCGCGGCATAGAACCGT												
repA (K02380)	F: AATTCAAACAACACTGTGCAGCCTG	7(14.6)	3(6.4)	0(0.0)	1(2.1)	0(0.0)	0(0.0)	0(0.0)	0(0.0)	0(0.0)	1(2.0)	8(16.3)	20(3.7)
	R: GCGAGAATGGACGATTACAAAACTTT												
Iterons (M20134)	F: CTATGGCCCTGCAAACGCGCCAGAAA	1(2.1)	0(0.0)	1(2.0)	0(0.0)	5(8.8)	2(5.6)	1(2.0)	1(2.8)	1(2.8)	1(2.0)	2(2.0)	15(2.8)
	R: TCACGCGCCAGGGCGCAGCC												
repA2 (AH003523)	F: GTGAACTGGCAGATGAGGAAGG	0(0.0)	0(0.0)	0(0.0)	0(0.0)	0(0.0)	0(0.0)	0(0.0)	0(0.0)	0(0.0)	2(3.9)	0(0.0)	2(0.4)
	R: TTCTCCTCGTCGCCAAACTAGAT												
repA (X73674)	F: GAGAACCAAAGACAAAGACCTGGA	0(0.0)	1(2.1)	1(2.0)	1(2.1)	0(0.0)	0(0.0)	0(0.0)	2(5.6)	1(1.4)	0(0.0)	1(2.0)	7(1.3)
	R: ACGACAAACCTGAATTGCCTCCTT												
repA (K00053)	F: TTGGCCTGTTTGTGCCTAAACCAT	0(0.0)	0(0.0)	0(0.0)	0(0.0)	0(0.0)	0(0.0)	0(0.0)	0(0.0)	0(0.0)	0(0.0)	0(0.0)	0(0.0)
	R: CGTTGATTACACTTAGCTTTGGAC												
repA (AE006471)	F: CTGTCGTAAGCTGATGGC	0(0.0)	0(0.0)	0(0.0)	0(0.0)	2(3.5)	0(0.0)	0(0.0)	0(0.0)	0(0.0)	0(0.0)	0(0.0)	2(0.4)
	R: CTCTGCCACAAACTTCAGC												
RNAI (AY234375)	F: TGATCGTTTAAGGAATTTTG	40(83.3)	40(85.1)	0(0.0)	0(0.0)	43(75.4)	0(0.0)	22(44.9)	28(77.8)	48(66.7)	36(70.6)	28(57.1)	285(52.6)
	R: GAAGATCAGTCACACCATCC												
RNAI (M93063)	F: CGGTCCGGAAAGCCAGAAAAC	0(0.0)	0(0.0)	1(2.0)	0(0.0)	0(0.0)	0(0.0)	0(0.0)	1(2.6)	0(0.0)	0(0.0)	0(0.0)	1(0.2)
	R: TCTTTCACGAGCCCGCCAAA												
RNAI (M28718)	F: GCGGTCCGGAAAGCCAGAAAAC	1(2.1)	0(0.0)	0(0.0)	0(0.0)	1(1.8)	0(0.0)	6(12.2)	2(5.6)	1(1.4)	1(2.0)	1(2.0)	13(2.4)
	R: TCTGCGTTCCGCCAAGTTCGA												

^a^Annealing position within the corresponding open reading frame (from the base A of start codon

ATG). ^b^ Y = T or C; R = A or G; S = G or C; D = A or G or T. ^c^ This primer pair was previously described.

**Figure 4.**
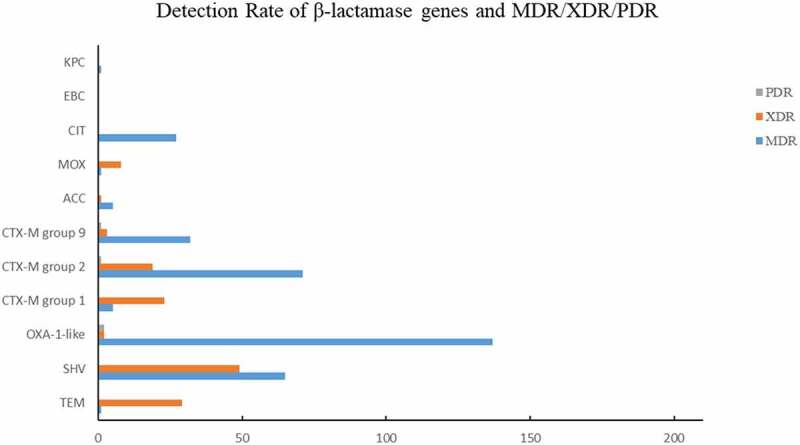
Detection Rate of β-lactamase genes and MDR/XDR/PDR.

Among the 378 MDR strains, *bla*_OXA-1_ acquired the highest carriage rate (137/378, 36.24%), followed by group 2 *bla*_CTX-M_ (71/378, 18.78%), *bla*_SHV-1_ (65/378, 17.20%), group 9 *bla*_CTX-M_ (32/378, 8.47%), and *bla*_CIT_ (27/378, 7.14%) (Figure 4). Among the 137 XDR strains, *bla*_OXA-1_ acquired the highest carriage rate (49/137, 35.77%), followed by *bla*_SHV-1_ (29/137, 21.17%), group 2 *bla*_CTX-M_ (23/137, 16.79%), and group 9 *bla*_CTX-M_ (19/137, 13.87%) (Figure 4). Among the 7 PDR strains, 2 strains carried *bla*_OXA-1_, 1 strains carried group 2 *bla*_CTX-M_ (23/137, 18.78%), and another one carried group 9 *bla*_CTX-M_, respectively.

### 3.5 Distribution of plasmid replicons

Eight groups of plasmid replicons were detected among the 542 *E. coli* isolates (Table 2). The *RNAI* (AY234375) in group 6 showed the highest detection rate (285/542, 52.58%), followed by the iteron (J01724, 233/542, 42.99%) and *repA* (M26308, 135/542, 24.91%) in group 3, indicating the relatively higher carriage rate of corresponding plasmids by clinical *E. coli* isolates. In group 1, *RNAI* (M20413) showed relatively higher detection rate (61/542, 11.25%), followed by *parA-parB* (AF250878, 31/542, 5.72%) and *iteron* (BX664015, 6/542, 1.11%). The identification rate of *RNAI* (M20413) was relatively stable among strains isolated in different years, except for in 2013 (none detected). In group 2, the identification rates of *oriγ* (Y00768), *repABC* (U27345), and *repA* (NC_003292) were 2.40%, 0.92%, and 4.43%, respectively. The *oriγ* (Y00768) was only detected in 2012 and 2018, while the *repABC* (U27345) was only detcted in 2009, 2011, and 2018. In group 3, except for iteron (J01724) and *repA* (M26308), *repA* (U12441) was carried by 135 (8.49%) *E. coli* strains isolated in 2012 and 2014–2016. The detection rate of the three genes in group 4 was relatively low, but the lowest identification rate was acquired by group 5. One of the repA genes (K00053) in group 5 was not detected. Only 1 strain isolated in 2010 carried the RNAI from group 7 (M93063).

**Figure 5.**
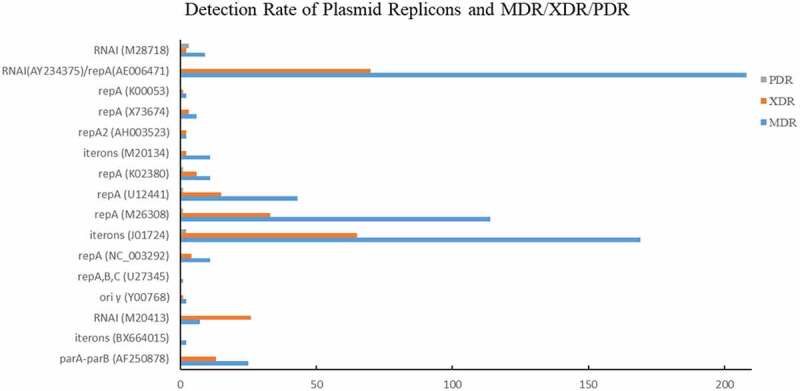
Detection Rate of Plasmid Replicons and MDR/XDR/PDR.

Among those MDR strains, the identification rates of *RNAI* (AY234375)/*repA* (AE006471), *iterons* (J01724), *repA* (M26308) were 55.03% (208/378), 44.71% (169/378), and 30.16% (114/378), respectively (Figure 5). Approximately 51.10% (70/137) and 47.45% (65/137) XDR strains carried *RNAI* (AY234375)/*repA* (AE006471) or *iterons* (J01724).

## 4. Discussion

In this study, females were more susceptible to *E. coli* infection, which is consistent with global trends that higher prevalence of urinary tract infections in female patients than in males [26–28]. Our result showed that urine was the main source of *E. coli*. Urinary tract infections (UTIs) caused by uropathogenic *Escherichia coli* (UPEC) are one of the most common outpatient bacterial infections [29]. The emergence of high rates of antibiotic resistance and multidrug-resistant MDR-phenotype from urinary tract infections related bacteria becomes a public health concern worldwide [30]. Moreover, the high resistance rates of penicillins (AMP) and fluoroquindones (LVX, CIP) during this period was in agreement with previous study [27,31].

Multidrug resistance has been increased all over the world that is considered a public health threat gave a warning to the potential and proper use of antibiotics [32,33]. Several recent investigations reported the emergence of multidrug-resistant bacterial pathogens from different origins including humans, birds, cattle, and fish that increase the need for routine application of the antimicrobial susceptibility testing to detect the antibiotic of choice as well as the screening of the emerging MDR strains [34–39]. The rate of MDR was higher than in other study from China, which reported only 47.9% of MDR strains [24]. It is known that susceptibility patterns may vary in diferent geographical regions and can be change over time. It is worth noting that MDR *E. coli* usually implies a signifcant increase in resistance and pathogenic potential and indicates complicated treatment and bad prognosis of infections [40]. Interestingly, resistance rates to antimicrobial drugs were higher in isolates from females than in those from male patients. Thus, it may be more difficult to eradicaten female infections and susceptibility analysis of isolates to antibiotics prior to treatment choice is recommended. For example, German national guideines agent as a first choice for the treatment of uncomplicated cystitis [30].

Over the last decade, *bla*_CTX-M_ has become increasingly common worldwide, to the point that their prevalence easily surpassing those of *bla*_SHV_ and *bla*_TEM_ ESBL genes [14]. Based on CTX-M amino acid sequences, these enzymes have been classified into five major groups, groups 1, 2, 8, 9 and 25. Previous study revealed that the incidence of these *bla*_CTX-M_ genotypes varies geographically. Our results showed that group 2 *bla*_CTX-M_ may be the most widely disseminated genotype in southern China, followed by group 9 and group 1.

Strains carrying *bla*_CIT_ were mainly isolated in 2010 and 2011. Six each group 1 *bla*_CTX-M_ and *bla*_AAC_ were detected, with group 1 *bla*_CTX-M_ identfied in strains isolated in 2008 and 2009, and *bla*_AAC_ carried by the strains isolated from 2010 to 2012. Two strains isolated in 2008 and 2011, respectively, carried *bla*_MOX_. Only one each strain isolated in 2011, 2018, and 2010 was identified to carry *bla*_TEM_, *bla*_EBC_, and *bla*_KPC_, respectively. In contrast, no group 8/25 *bla*_CTX-M_, *bla*_AAC_, *bla*_FOX_, *bla*_DHA_, *bla*_GES_, *bla*_PER_, *bla*_VEB_, *bla*_OXA-48_, *bla*_IMP_, and *bla*_VIM_ genes were identfied.

Among the 94 isolates that were resistant to β-Lactam, surprisingly 64 did not have any of the 21 β-lactam resistance genes. These results suggested that there may be no relation between the presence of *bla*_TEM_, *bla*_SHV_, *bla*_OXA-1-like_, *bla*_CTX-M_, *bla*_ACC_, *bla*_MOX_, *bla*_CIT_, *bla*_EBC_, and *bla*_KPC_ with β-lactam production and other types of β-lactam resistant genes may be responsible for the phenotype. More importantly, the expression of β-lactam genes depends upon the environmental conditions such as the presence of antibiotics and genes presence shown by PCR does not necessarily indicate its expression.

Plasmids are extra-chromosomal circular fragments of DNA that replicate autonomously in *E. coli*. Plasmids appear to increase bacterial genetic diversity, containing genes essential for initiation and control of replication and accessory genes that may be useful to their bacterial host such as antimicrobial resistance or virulence genes [41]. Since the origin of replication is a constant and conserved part of a plasmid, replicon typing focused on this portion of the plasmid is a more sensitive and specific method for identifying phylogenetically related plasmids than restriction-based analysis of the entire plasmid. The classfication of plasmid is usually based on PCR-based replicon typing (PBRT) method which relied on the phenomenon of incompatibility that closely related plasmids cannot coexist stably in the same cell [42].

Plasmids are significantly correlated with bacterial pathogenicity. IncFI (pEM4) and IncI (pEM6) are found to determine virulence and colicine I production and resistance to tetracycline [43]. *RNAI*, a small countertranscript RNA which can inhibit translation of *repA* mRNA, expressed the incompatibility of IncI plasmids in the PBRT scheme. IncI plasmids generate both a thick pilus (*tra* genes) for DNA transfer and athin pilus (*pil* genes) that appears to stabilize the mating apparatus in liquid media but not on solid surfaces, which is reported to result in different conjugation efficiencies and biofilm and/or adherence properties [44,45]. ESBL and plasmid-mediated (p)AmpC genes have been described on IncI plasmids in *E. coli. bla*_CTX-M-1_ is the most often identified gene on IncI plasmid, followed by *bla*_CMY-2_ and *bla*_CTX-M-15_ [46]. *RepA*, broadly distributed throughout the low G + C Gram-positive bacteria, is the target site of IncA/C, IncN, IncF and IncT plasmid In the PBRT (PCR-based replicon typing) scheme. Incompatibility group IncA/C plasmids are large, low copy plasmids that associated with enteric pathogens of humans and animals [47,48] IncA/C plasmids are associated with MDR and can encode ESBLs (*bla*_TEM_, *bla*_SHV_, but rarely *bla*_CTX-M_), AmpC (bla_CMY_, bla_DHA_), carbapenemases (*bla*_OXA_, *bla*_NDM_, *bla*_IMP_) and enzymes modifying groups of antibiotics: sulfonamides (*sul1, sul2*), aminoglycosides (*aphA1, aadA, aadB, strA, strB, aacC*), tetracyclines, chloramphenicol (*floR*, catA1) and trimethoprim (*dfrA*) [49,50]. IncN plasmids carry a great variety of resistance determinants against ESBL, sulfonamides, quinolones, aminoglycosides, tetracyclines and streptomycin [51]. The most frequently described resistance genes on IncF plasmids are ESBL genes (genes encoding carbapenemases, aminoglycoside-modifying enzymes and plasmid-mediated quinolone resistance (PMQR) genes). IncF plasmids also drive the spread of *bla*_NDM_ and the *rmtB* gene (mostly reported in China) [52]. IncT plasmids usually carry kanamycin- (*Rst1*) or sulfonamide resistance genes [53]. *ParA-parB* is the target site for typing IncHI plasmids in the PBRT scheme, which has been reported to contain large virulence plasmid pWR100 [54]. IncH plasmids are reported to be associated with multidrug resistance because, besides ESBL genes, they often carry genes encoding for resistance to sulfonamides, aminoglycosides, tetracyclines and streptomycin [55]. IncP is a group of broad-host-range, low-copy-number plasmids, the copy number of which is controlled by *iterons* [56]. A recurring theme in the duplication of prokaryotic replicons is the recognition of the replication origin by cis-encoded initiators that bind to repeated nucleotide sequences called *iterons*. They were reported to carry genes conferring resistance to ESBL, sulfonamides, aminoglycosides and tetracyclines [57,58]. *Oriγ* is the target site of IncX, a group of narrow-host-range plasmids encoding primarily AMR determinants against ESBL and quinolones. These plasmids encode primarily AMR determinants against ESBL and quinolones [59,60]. Its six known subtypes (X1-X6) were releveant to tetracycline and trimethoprim resistance determinants. IncX plasmids were able to form cointegrants with Salmonella serotype-specific plasmid-carrying virulence genes which resulted in a broadening of the host range of the new plasmid [61].

## 5. Conclusions

This study had designed and developed multiplex amplification platform for rapid and accurate detection of such resistance genes in 542 clinical *E. coli* isolates. It was found that most MDR isolates were co-resistant to penicillins (AMP) and fluoroquindones (LVX, CIP) and distribution of LVX and CIP resistance was significantly higher among female than male gender. Among the β-lactamase genes, *bla*_OXA_ acquired the highest carriage rate, followed by group 2 *bla*_CTX-M_ and *bla*_SHV-1_, indicating their prevalence among clinical *E. coli* in South China. The *RNAI* (AY234375) showed the highest detection rate, followed by the *iteron* (J01724) and *repA* (M26308), indicating the relatively higher carriage rate of corresponding plasmids by clinical *E. coli* isolates. A high percentage of the isolated *E. coli* strains were multidrug resistant (MDR) to penicillins: ampicillin, aminoglycosides, and fluoroquinolones; and are harboring the *bla*_OXA-1_, group 2 *bla*_CTX-M_, and *bla*_SHV-1_ genes, as well as *RNAI*(AY234375)/*repA*(AE006471), *iterons* (J01724) genes. It is shown that such identification of plasmid replicons and β-lactamase genes may aid in the understanding of clinical *E. coli* isolates epidemiology. A strategy design based on multiplex amplification and further application for rapid detection on antimicrobial resistance and β-lactam resistance genes in clinical *Escherichia coli* strains.

## Data Availability

All data generated or analyzed during this study are included in this article. The authors confirm that the data supporting the findings of this study are available within the article
